# The burden among caregivers who are caring for patients with dementia admitted under private nursing services

**DOI:** 10.3389/frdem.2026.1873575

**Published:** 2026-09-11

**Authors:** Asmahan A. M. Abdo, Erilyn Leigh C. Manalo, Liela Santander, Janice Samaco, Myra Aler, Moby Benny, Deborah Okemmuo, Maria Katrina Bacal, Samah Swedan, Kalpana Singh

**Affiliations:** 1 Private Nursing Services, Hamad Medical Corporation, Doha, Qatar; 2 Nursing Department, Hamad Medical Corporation, Doha, Qatar

**Keywords:** caregiver burden, caregiver support systems, dementia, emotional strain, private nursing services

## Abstract

**Background:**

The increasing prevalence of dementia, particularly among aging populations, presents significant challenges for caregivers.

**Objective:**

This study explores the burden experienced by caregivers of dementia patients under Private Nursing Services (PNS) in Qatar, emphasizing the physical and emotional burden.

**Methods:**

A descriptive cross-sectional design was employed, involving 101 caregivers. The Zarit Burden Interview (ZBI), a 22-item multidimensional tool, assessed caregiver burden across physical, emotional, and financial domains. Sociodemographic data such as age, gender, education, and caregiving hours were also collected.

**Results:**

Findings revealed that 36.6% of caregivers experienced moderate to severe burden, with the highest strain reported by spouses and individuals with lower education levels. Female caregivers constituted 75.2% of the participants, and those dedicating over 16–24 h daily to caregiving reported intense burden. Key stressors included emotional strain, lack of personal space, health concerns, and financial instability. The average ZBI score of 42.8 ± 19.6 indicates moderate to severe caregiving challenges.

**Conclusion:**

The study underscores the need for targeted interventions, such as mental health support, caregiver education, and training programs, to alleviate the burden and enhance the well-being of caregivers. These findings highlight the importance of comprehensive support systems, including respite care, counseling, and educational workshops, to improve the quality of life for both caregivers and dementia patients. Future research should examine shared caregiving responsibilities and explore strategies to address logistical challenges in caregiving contexts.

## Introduction

Dementia is rapidly emerging as one of the most significant public health challenges of the 21st century, with its global prevalence doubling approximately every 20 years due to an aging population. This alarming trend underscores the pressing need for comprehensive strategies to mitigate its impact on individuals, families, and healthcare systems worldwide (Alzheimer’s Disease International, 2016). Dementia is characterized by progressive deterioration in memory, cognitive function, and behavior, significantly impairing individuals’ ability to manage everyday activities. Beyond the affected individuals, the burden extends to caregivers and families, often leaving them grappling with physical, emotional, and financial challenges. In response to these challenges, the World Health Organization’s (WHO) Global Action Plan on the Public Health Response to Dementia (2017–2025) provides a framework aimed at achieving physical, mental, and social well-being for both dementia patients and their caregivers. Central to this framework is the recognition of the pivotal role caregivers play in supporting individuals with dementia and the need for tailored interventions to enhance their quality of life.

Caregivers’ perspectives are essential in understanding the multifaceted challenges associated with dementia care. Their insights illuminate the complexities of caregiving roles and can guide the development of targeted strategies to improve caregiving experiences. Addressing caregivers’ needs and perceptions is crucial, as these factors significantly influence both their quality of life and the care provided to dementia patients. Domínguez Vergara et al. (2023) emphasized that caregivers of individuals with intellectual disabilities, including dementia, often experience profound psychological distress due to the demanding nature of their responsibilities. The progressive nature of dementia exacerbates these challenges, making caregiver support an integral component of dementia care strategies.

In Qatar, the burden of dementia caregiving is particularly pronounced. A retrospective study identified Alzheimer’s disease and vascular dementia as the most prevalent types among home care patients, with hypertension and atherosclerosis being the leading contributing factors (Sulaiti et al., 2008). Depression frequently emerged as a common comorbidity among dementia patients, adding to the psychosocial burden experienced by caregivers. Despite these challenges, Qatar has taken proactive measures to address the needs of caregivers through comprehensive support systems. The Qatar National Dementia Plan (2018) (2018–2022) and initiatives led by the Qatar Alzheimer’s Society aim to position dementia as a national priority, raise awareness, and provide robust support mechanisms for caregivers. These efforts highlight the importance of empowering caregivers and equipping them with the resources needed to navigate their roles effectively.

Currently, Private Nursing Services (PNS) in Qatar provide care for 146 dementia patients, underscoring the critical role of caregivers in meeting the escalating needs of this population. However, caregivers often bear the brunt of these responsibilities at the expense of their physical, emotional, and financial well-being. The intense demands of caregiving frequently result in psychological distress, manifesting as guilt, resentment, and depression. The multifaceted nature of caregiver burden necessitates comprehensive evaluation tools, such as the Zarit Burden Interview (ZBI), which assesses the impact of caregiving on caregivers’ health, relationships, and finances. The ZBI has demonstrated robust internal consistency across diverse settings, making it a valuable tool for understanding the nuances of caregiver burden (Springate and Tremont, 2014).

This study aims to evaluate the burden experienced by caregivers of dementia patients under PNS in Qatar and to explore the associations between this burden and various demographic and psychological factors. By examining these dimensions, the research seeks to inform strategies to alleviate caregiver strain and enhance the quality of life for both caregivers and dementia patients.

## Methodology

### Study design and sampling

This descriptive cross-sectional study aims to evaluate the burden faced by caregivers of dementia patients in Qatar. The study utilized the Zarit Burden Interview (ZBI) survey tool to measure caregiver burden across physical, emotional, social, and financial aspects. Additionally, it explored the association between caregiver burden and sociodemographic factors such as age, gender, educational background, and caregiving frequency.

### Population and setting

The study targeted caregivers of dementia patients receiving nursing services under the PNS program at Hamad Medical Corporation (HMC). A total of 146 participants were recruited. Caregivers were defined as individuals dedicated to providing care for dementia patients, including family members, friends, or paid/unpaid caregivers. Data collection took place in patients’ homes across Qatar. Inclusion criteria include primary caregivers actively involved in the daily care of dementia patients under PNS who can read and complete an English survey. Exclusion criteria was caregivers whose patients are not under PNS care, caregivers not handling dementia patients, and those who do not understand English.

### Data collection tool

The Zarit Burden Interview (ZBI) is a validated 22-item global measure of caregiver burden. While it produces a single overall burden score, individual items reflect different aspects of caregiving strain, such as emotional, physical, social, and financial challenges. In this study, we report the global burden score and use item-level responses to illustrate the types of difficulties most frequently experienced by caregivers. According to Al-Rawashdeh et al. (2016), the ZBI’s reported Cronbach’s alpha ranges from 0.85 to 0.93 in dementia caregiving contexts, indicating high reliability.

The ZBI does not include predefined sections but identifies thematic dimensions through factor analysis, such as emotional, physical, and social tolls; relationship strain; emotional well-being; financial impact; and loss of personal freedom. Responses are scored on a Likert scale (0–4), with the following levels: (0): Never burdened, (1): Mild burden, (2): Moderate burden, (3): Severe burden, (4): Extreme burden;

The total score ranges from 0 to 88, with higher scores indicating a greater perceived burden.

The survey was self-administered in English via a digital tablet, taking 10–15 min to complete. The survey was collected sociodemographic data and include the ZBI to assess caregiver burden. As part of the survey, the caregiver was asked to provide sociodemographic information including age, gender, relationship with the patient (partners, children, etc.), level of education, whether he or she lived with the patient, number of contact hours per week with the patient, and patient health status.

### Sample size

This study was designed as a census of all caregivers under the Private Nursing Services program. A total of 146 caregivers were approached, and 101 completed the Zarit Burden Interview survey in full. Since the study encompassed the entire available caregiver population, a formal sample size calculation was not performed. The study encompassed all caregivers providing care for patients with dementia. A caregiver was defined as an individual fully committed to delivering care for dementia patients. This included family members, friends, and both paid and unpaid caregivers. These individuals played a central role in managing the daily needs and overall well-being of the dementia patients under their care.

Caregivers were recruited through the Private Nursing Services program at Hamad Medical Corporation. All registered caregivers were approached during routine visits and invited to participate. Participation was voluntary, and informed consent was obtained prior to data collection. Of the 146 caregivers approached, 101 completed the Zarit Burden Interview survey, yielding a response rate of 69.1%. Caregivers who declined participation or provided incomplete responses were excluded from the analysis.

### Statistical analysis

The data were prepared and analyzed using STATA 17.0. The preparation process involved cleaning and organizing the dataset to ensure consistency, accuracy, and completeness. Descriptive statistics were employed to summarize the sociodemographic characteristics of the participants and their scores on the Zarit Burden Interview (ZBI). To see the relationships between caregiver burden and sociodemographic factors, one-way analysis of variance (ANOVA) was used to identify significant differences in ZBI scores across and years of taking care of their children’s, Chi square test was used to see the statistical difference between the categorical variables. Assumptions of ANOVA (normality and homogeneity of variance) were checked prior to analysis. The results of these statistical analyses provided insights into the patterns and predictors of caregiver burden. Significance levels were set at *p* < 0.05, and all tests were two-tailed.

## Results

The study included 101 participants, with an average age of 43.2 ± 11.5 years. The majority were female (75.2%). Most participants were either Arab (55.5%) or Asian (44.6%). In terms of education, with 45% being graduates, 33% completing secondary education, and smaller proportions having primary education (14%) or being illiterate (8%). Most caregivers (67%) lived with other family members, while 33% resided solely with the care recipient. Caregiving commitments were intense, with 43.6% dedicating 16–24 h daily and a mean caregiving duration of 5.6 ± 4.8 years (Table 1).

**Table 1:** Participant characteristics.

Variables	Level	Value
*N*		101
Age in Years, mean (SD)		43.2 (11.5)
Gender	Female	76 (75.2%)
	Male	25 (24.8%)
Nationality	Asian	45 (44.6%)
	Arabs	55 (55.5%)
Education Attainment	Graduate or above	45 (45.0%)
	Illiterate	8 (8.0%)
	Primary School	14 (14.0%)
	Second School	33 (33.0%)
Relationship to the care recipient	Brother /Sister	6 (5.9%)
	Brother-in-Law /Sister-in-Law	2 (2.0%)
	Others	47 (46.5%)
	Son/ Daughter	33 (32.7%)
	Spouse	13 (12.9%)
Current living arrangements	Jointly with other family member	67 (67.0%)
	Only with care recipients (patient)	33 (33.0%)
How many hours per day do you spend caregiving?	0–8	23 (22.8%)
	16–24	44 (43.6%)
	8–16	34 (33.7%)
Taking care of this patient in Years, mean (SD)		2.6 (4.8)

Table 2 showed the caregiver burden scale various levels of challenges faced by caregivers. Each response corresponds to the following levels: 0- Never burden at all, 1- Mild burden, 2- Moderate burden, 3- Severe burden and 4- Extreme burden. A significant proportion reported insufficient personal time (34% at level 3) and feeling overburdened with responsibilities (35% at level 2). Many experienced a moderate loss of control over their lives (26% at level 3) and uncertainty about caregiving (31% at level 3). Caregivers often felt they should do more (29% at level 3) or could improve (32% at level 4). Emotional strain was common (23% at level 2), while anger and embarrassment were less significant, with 45 and 44% reporting none, respectively. Social life and relationships were moderately affected, with caregiving straining connections and privacy (21% at level 4). Health concerns (22% at level 4), excessive help requests (29% at level 3), and unequal caregiving responsibilities (21% at level 4) highlighted the physical and emotional toll. Fear of the future (30% at level 2), financial worries (22% at level 3), and doubts about sustaining care (22% at level 3) were prevalent. Although 35% did not wish to delegate care, many reported significant dependence from care recipients (37% at level 4). Overall, the average burden score of 42.8 ± 19.6 reflects moderate to severe caregiving challenges with 36.6% experiencing moderate-to-severe levels and 18.8% facing severe caregiving challenges (Table 2).

**Table 2:** Assessment of Caregiver Burden Scale across multiple dimensions.

Variables	Level	Value
*N*		101
There is not enough time for yourself	Never burden at all	7 (7%)
	Mild burden	16 (16%)
	Moderate burden	27 (27%)
	Severe burden	34 (34%)
	Extreme burden	15 (15%)
Overtaxed with responsibilities	Never burden at all	8 (8%)
	Mild burden	10 (10%)
	Moderate burden	34 (35%)
	Severe burden	28 (29%)
	Extreme burden	17 (18%)
Like you have lost control over your life	Never burden at all	16 (16%)
	Mild burden	21 (21%)
	Moderate burden	23 (23%)
	Severe burden	26 (26%)
	Extreme burden	13 (13%)
Uncertain about what to do for your relative	Never burden at all	12 (13%)
	Mild burden	16 (17%)
	Moderate burden	25 (26%)
	Severe burden	30 (31%)
	Extreme burden	13 (14%)
Like you should do more for your relative	Never burden at all	6 (6%)
	Mild burden	18 (18%)
	Moderate burden	25 (26%)
	Severe burden	28 (29%)
	Extreme burden	21 (21%)
Like you could do a better job of caring	Never burden at all	5 (5%)
	Mild burden	10 (10%)
	Moderate burden	24 (24%)
	Severe burden	28 (29%)
	Extreme burden	31 (32%)
A sense of strain	Never burden at all	20 (21%)
	Mild burden	19 (20%)
	Moderate burden	22 (23%)
	Severe burden	19 (20%)
	Extreme burden	17 (18%)
Anger	Never burden at all	45 (45%)
	Mild burden	14 (14%)
	Moderate burden	16 (16%)
	Severe burden	15 (15%)
	Extreme burden	9 (9%)
Embarrassment	Never burden at all	44 (44%)
	Mild burden	15 (15%)
	Moderate burden	14 (14%)
	Severe burden	16 (16%)
	Extreme burden	10 (10%)
Uncomfortable about having friends over	Never burden at all	37 (38%)
	Mild burden	18 (18%)
	Moderate burden	19 (19%)
	Severe burden	15 (15%)
	Extreme burden	9 (9%)
Your social life	Never burden at all	31 (31%)
	Mild burden	15 (15%)
	Moderate burden	26 (26%)
	Severe burden	14 (14%)
	Extreme burden	13 (13%)
Other relationships with family and friends	Never burden at all	27 (28%)
	Mild burden	16 (16%)
	Moderate burden	20 (20%)
	Severe burden	22 (22%)
	Extreme burden	13 (13%)
Your health	Never burden at all	18 (18%)
	Mild burden	12 (12%)
	Moderate burden	24 (24%)
	Severe burden	22 (22%)
	Extreme burden	22 (22%)
Your privacy	Never burden at all	25 (25%)
	Mild burden	15 (15%)
	Moderate burden	18 (18%)
	Severe burden	20 (20%)
	Extreme burden	21 (21%)
Feel you receive excessive help requests	Never burden at all	11 (11%)
	Mild burden	16 (16%)
	Moderate burden	25 (25%)
	Severe burden	29 (29%)
	Extreme burden	18 (18%)
Feel all the responsibility falls on one caregiver	Never burden at all	19 (19%)
	Mild burden	14 (14%)
	Moderate burden	26 (27%)
	Severe burden	18 (18%)
	Extreme burden	21 (21%)
Fear the future regarding your relative	Never burden at all	24 (25%)
	Mild burden	9 (9%)
	Moderate burden	29 (30%)
	Severe burden	17 (18%)
	Extreme burden	17 (18%)
Fear not having enough money to care for your relative	Never burden at all	27 (27%)
	Mild burden	8 (8%)
	Moderate burden	25 (25%)
	Severe burden	22 (22%)
	Extreme burden	17 (17%)
Fear not being able to continue caring for your relative	Never burden at all	28 (28%)
	Mild burden	16 (16%)
	Moderate burden	18 (18%)
	Severe burden	22 (22%)
	Extreme burden	15 (15%)
Wish to leave the care of your relative to someone else	Never burden at all	35 (35%)
	Mild burden	9 (9%)
	Moderate burden	29 (29%)
	Severe burden	13 (13%)
	Extreme burden	13 (13%)
How much does your loved one depend on you as the caregiver?	Never burden at all	5 (5%)
	Mild burden	8 (8%)
	Moderate burden	23 (23%)
	Severe burden	26 (26%)
	Extreme burden	37 (37%)
Burden score, mean (SD)		42.8 (19.6)
Caregivers burden	Minimal burden	15 (14.9%)
	Moderate burden	30 (29.7%)
	Moderate to severe burden	37 (36.6%)
	Severe burden	19 (18.8%)

The Table 3 showed significant differences in caregiver burden based on education level and relationship to the care recipient. Regarding education, 73% of caregivers with graduate or higher education experienced minimal burden, compared to only 26% of those with secondary education, indicating that higher educational was associated with a lower caregiver burden. In contrast, 49% of those with secondary school education and 42% with illiteracy reported moderate to severe burden. As for relationship to the care recipient, 37% of spouses experienced severe burden, while 11% of siblings and 16% of other relatives reported similar levels of burden.

**Table 3:** Association between Caregiver Burden Scale and demographic and caregiving factors.

Variables	Level	Minimal burden	Moderate burden	Moderate to severe burden	Severe burden	*p*-value
*N*		15	30	37	19	
Age in Years, mean (SD)		42.0 (15.0)	40.8 (10.0)	44.5 (11.4)	45.5 (10.8)	0.47
Gender	Female	12 (80%)	20 (67%)	28 (76%)	16 (84%)	0.53
	Male	3 (20%)	10 (33%)	9 (24%)	3 (16%)	
Nationality	Asia	5 (45%)	11 (46%)	13 (41%)	7 (37%)	0.93
	Arab	6 (55%)	13 (54%)	19 (59%)	12 (63%)	
Education	Graduate or above	11 (73%)	19 (66%)	10 (27%)	5 (26%)	0.019
	Illiterate	0 (0%)	2 (7%)	3 (8%)	3 (16%)	
	Primary school	2 (13%)	3 (10%)	6 (16%)	3 (16%)	
	Second school	2 (13%)	5 (17%)	18 (49%)	8 (42%)	
Relationship to the care recipient	Brother/sister	0 (0%)	3 (10%)	1 (3%)	2 (11%)	0.011
	Brother-in-law/sister-in-law	0 (0%)	0 (0%)	1 (3%)	1 (5%)	
	Others	8 (53%)	17 (57%)	19 (51%)	3 (16%)	
	Son/daughter	7 (47%)	10 (33%)	10 (27%)	6 (32%)	
	Spouse	0 (0%)	0 (0%)	6 (16%)	7 (37%)	
Current living arrangements	Jointly with other family member	7 (47%)	18 (62%)	26 (70%)	16 (84%)	0.12
	Only with care recipients (patient)	8 (53%)	11 (38%)	11 (30%)	3 (16%)	
Hours per day do you spend caregiving	0–8	3 (20%)	9 (30%)	8 (22%)	3 (16%)	0.32
	16–24	6 (40%)	11 (37%)	14 (38%)	13 (68%)	
	8–16	6 (40%)	10 (33%)	15 (41%)	3 (16%)	
Years of taking care of this patient, mean (SD)		4.4 (3.3)	5.5 (4.7)	5.2 (4.2)	7.7 (6.5)	0.20

The variables, such as age, gender, nationality, current living arrangements, caregiving hours per day, and years of caregiving, no significant differences in caregiver burden scale were observed across different levels (Table 3).

## Discussion

This study highlights the substantial burden experienced by caregivers of dementia patients, with 36.6% reporting moderate to severe levels of burden. This finding is clinically significant and mirrors results from recent studies in Saudi Arabia, Kuwait, and the UAE, which also report high emotional and financial strain among dementia caregivers (Alqahtani et al., 2023; Al Kuwari et al., 2024). These regional comparisons emphasize that caregiver burden is a shared challenge across Gulf countries and requires coordinated policy responses. These findings underscore the significant impact caregiving has on caregivers’ well-being, necessitating targeted interventions to mitigate this burden and improve caregivers’ quality of life.

The study reveals that educational attainment is a crucial determinant of caregiver burden. Caregivers with graduate or higher education reported significantly lower burden levels compared to those with secondary or lesser education. This disparity suggests that higher education may equip caregivers with better problem-solving skills, emotional resilience, and access to support networks. These findings align with prior studies suggesting that education enhances caregivers’ ability to navigate challenges effectively (Schulz and Sherwood, 2008; Chiao et al., 2015). Recent Middle Eastern research also supports this, showing that caregivers with higher education levels are more likely to access formal support services and demonstrate lower psychological distress (Al Harthi et al., 2025).

Spouses of dementia patients were found to experience the highest levels of severe burden, with 37% reporting severe caregiving challenges. This finding underscores the emotional and relational toll spousal caregiving entails. Spouses often bear the primary responsibility for caregiving, leading to heightened emotional distress, role strain, and isolation. These results echo previous research demonstrating that spousal caregivers face unique challenges, including loss of companionship and increased dependency of the care recipient (Pinquart and Sörensen, 2011). Cultural expectations in Gulf societies, where spousal and family caregiving is considered a moral and religious duty, further intensify this burden (Hammad et al., 2024; Thompson et al., 2024).

Caregivers dedicating 16–24 h daily to caregiving reported higher levels of burden, highlighting the risk of caregiver burnout. This intensive time commitment is often unsustainable and negatively affects caregivers’ physical and mental health. Respite care, flexible caregiving schedules, and support services could help alleviate these burdens by providing caregivers with much-needed breaks and opportunities for self-care. Similar findings have been reported in UAE and Saudi studies, where continuous caregiving hours were strongly associated with caregiver depression and anxiety (Alqahtani et al., 2023).

The study revealed that many caregivers experience emotional strain, uncertainty, and a sense of loss of control in their roles. Feelings of inadequacy and the desire to do more for the care recipient were common. Social life and relationships were also strained, with caregivers reporting reduced privacy and fewer opportunities for social engagement. These findings emphasize the need for emotional support programs, such as counseling and peer support groups, to foster social connections and reduce feelings of isolation. Recent global research highlights the effectiveness of psychosocial interventions and mHealth tools in reducing caregiver stress, suggesting that such approaches could be adapted to the Qatari context (Meng et al., 2025).

The physical and mental health of caregivers was significantly affected, with many reporting health deteriorations due to caregiving responsibilities. Additionally, financial concerns were prevalent, with 22% of caregivers expressing fears about not having sufficient resources to sustain care. These findings highlight the importance of integrated support systems that address caregivers’ health and financial well-being, including access to healthcare services, financial counseling, and subsidies for caregiving expenses. This is consistent with regional evidence showing that financial strain is one of the most pressing challenges for dementia caregivers in the Middle East.

### Recommendations

The findings highlight an urgent need to strengthen comprehensive support systems for caregivers through a multi-faceted approach. This includes implementing structured educational interventions such as workshops and training programs to enhance caregivers’ knowledge and practical skills, alongside the development of accessible respite care services that provide temporary relief through professional support. Equally important is the establishment of counseling services and peer support groups to address emotional strain and improve relational well-being. Financial assistance programs, including subsidies and financial planning support, are essential to reduce the economic burden on caregiving households. In the context of Qatar, these recommendations should be integrated into the existing healthcare infrastructure, particularly through the Ministry of Public Health and Hamad Medical Corporation. Respite care services could be expanded via the Private Nursing Services program, caregiver education workshops can be delivered through primary healthcare centers, and counseling services should be embedded within community health facilities. Financial subsidies and planning support should be incorporated into Qatar’s National Dementia Plan to ensure sustainability and accessibility for caregiving households. Furthermore, integrating caregiver-focused services within the healthcare system will ensure timely access to both physical and mental health resources, ultimately promoting caregiver well-being and improving the quality of care provided.

#### Policy implications

The findings highlight the urgent need for policy changes that recognize and address the challenges faced by caregivers. This may include advocating for increased funding for caregiver support programs, creating flexible work policies, and enhancing training for healthcare professionals regarding caregivers’ needs. Specifically, within Qatar’s policy environment, caregiver support should be prioritized in the National Dementia Plan and aligned with the country’s broader health strategy. Increased government funding could be directed toward expanding respite care and mental health services, while flexible work policies should be developed in collaboration with the Ministry of Labor to support employed caregivers. Training modules for healthcare professionals should be mandated by Hamad Medical Corporation to ensure that caregiver needs are systematically addressed in clinical practice.

### Limitation

This study has several limitations. The cross-sectional design precludes causal inference, and reliance on self-report introduces potential bias. Generalizability is limited due to the small sample size and lack of longitudinal assessment. Additional limitations include possible selection bias from English-language survey administration, nonresponse bias, and the absence of adjustment for dementia severity, behavioral symptoms, and patient functional status. Furthermore, caregiver resilience, coping styles, and social support were not assessed, although these are established predictors of caregiver burden. Future studies should incorporate these variables to provide a more comprehensive understanding of caregiver experiences.

## Conclusion

This study demonstrates that dementia caregiving in Qatar imposes a substantial burden, particularly among spousal caregivers and those with lower educational attainment. These findings highlight the urgent need for comprehensive support services, including respite care, caregiver education, mental health support, and financial assistance programs. By integrating caregiver-focused interventions into Qatar’s healthcare infrastructure and aligning them with the National Dementia Plan, policymakers can ensure culturally appropriate and sustainable support. While the study’s cross-sectional design limits causal inference, the results provide valuable insights into caregiver experiences and underscore the importance of targeted interventions to improve caregiver well-being and patient care outcomes.

## Data Availability

The raw data supporting the conclusions of this article will be made available by the authors, without undue reservation.
